# Surgery in rare bleeding disorders: the prospective MARACHI study

**DOI:** 10.1016/j.rpth.2023.102199

**Published:** 2023-09-06

**Authors:** Florence Rousseau, Benoit Guillet, Thibault Mura, Alexandra Fournel, Fabienne Volot, Hervé Chambost, Pierre Suchon, Brigit Frotscher, Christine Biron-Andréani, Raphaël Marlu, Nathalie Hezard, Ségolène Clayssens, Elodie Boissier, Florence Blanc-Jouvan, Pierre Chamouni, Nathalie Tieulie, Lucia Rugeri, Annie Borel-Derlon, Emmanuelle de Raucourt, Isabelle Martin-Toutain, Sabine Castet, Aurélien Lebreton, Stéphane Girault, Dominique Helley-Russick, Roseline D’Oiron, Jean-François Schved, Muriel Giansily-Blaizot

**Affiliations:** 1 Département d’hématologie biologique, CHU Montpellier, France; 2 CRC-MHC, CHU Montpellier, France; 3 Haemophilia Treatment Center, University Hospital, Rennes, France and Univ Rennes, CHU Rennes, Inserm, EHESP, Irset (Institut de recherche en santé, environnement et travail) - UMR_S 1085, F-35000 Rennes, France; 4 Département d’informatique médicale, CHU Montpellier, CHU Nîmes, Université de Montpellier FR 34090, France; 5 Haemophilia Treatment Centre, University Hospital of Besançon, Besançon, France; 6 CRC-MHC Dijon, CHU Dijon, France; 7 AP-HM Marseille, France; 8 Aix Marseille University, Inserm, Inrae, C2VN, Marseille, France; 9 Haemophilia Treatment Centre, University Hospital of Nancy, France; 10 Hemostasis Unit, CHU Grenoble Alpes, Université Grenoble Alpes, France; 11 Laboratoire d’hématologie, CHU Reims, France; 12 Laboratoire d’hématologie AP-HM Marseille, France; 13 CRC-MHC, CHU Toulouse, France; 14 Laboratoire d’hématologie, CHU Nantes, France; 15 Laboratoire d’hématologie, CH Annecy Genevois, France; 16 HTC, University Hospital, Rouen, France; 17 Service de Rhumatologie, CHU Nice, France; 18 Unité Hémostase Clinique, Hospices Civils de Lyon, France; 19 CRC-MHC, CHU Caen, France; 20 Service d’hématologie Biologique Hôpital Beaujon, HUPNVS, AP-HP, France; 21 CRC-MHC, Service d’hématologie clinique, Hôpital Mignot Le Chesnay, France; 22 CRC-MHC, CHU Bordeaux, France; 23 Laboratoire d’hématologie, CHU Clermont Ferrand, Unité de Nutrition Humaine UMR1019, INRAE / Université Clermont Auvergne, Clermont-Ferrand, France; 24 Consultation d’hémostase, CHU Limoges, France; 25 Laboratoire d’hématologie, Hôpital Georges Pompidou, AP-HP, France; 26 Centre de référence de l’hémophilie et des maladies hémorragiques constitutionnelles, Hôpital Bicêtre, APHP and Hith, UMR_S1176, INSERM, Université Paris Saclay, Le Kremlin Bicêtre, France; 27 Université Montpellier d’excellence, Montpellier, France

**Keywords:** bleeding score, hemorrhagic disorders, rare bleeding disorder, replacement therapy, surgery, tranexamic acid

## Abstract

**Background:**

Despite the wide use of bleeding scores and the reliability of clotting factor level measurement, bleeding risk stratification before surgery remains challenging in patients with rare inherited bleeding disorders.

**Objectives:**

This multicenter observational prospective study assessed in patients with rare coagulation factor deficiency, the perioperative hemostatic management choices by hemostasis experts and the bleeding outcomes after surgery.

**Methods:**

One hundred seventy-eight patients with low coagulation activity level (factor [F] II, FV, combined FV-FVIII, FVII, FX, or FXI <50%) underwent 207 surgical procedures. The bleeding outcome, Tosetto’s bleeding score, and perioperative hemostatic protocols were collected.

**Results:**

Among the 81 procedures performed in patients with severe factor deficiency (level ≤10%), 27 were done without factor replacement (including 6 in patients at high bleeding risk), without any bleeding event. Factor replacement therapy was used mainly for orthopedic procedures. In patients with mild deficiency, 100/126 surgical procedures were carried out without perioperative hemostatic treatment. In patients with FVII or FXI deficiency, factor replacement therapy was in function of the procedure, bleeding risk, and to a lesser extent previous bleeding history. Tranexamic acid was used in almost half of the procedures, particularly in case of surgery in tissues with high fibrinolytic activity (76.8%).

**Conclusions:**

The current perioperative hemostatic management of patients with rare bleeding disorders appears to be adapted. Among the 207 procedures, only 6 were associated with excessive bleeding. Our findings suggest that rather than the bleeding score, factor level and surgery type are the most relevant criteria for perioperative factor replacement therapy.

## Essentials

• Surgical bleeding risk management is challenging in rare bleeding disorders.
• This multicenter, prospective study evaluated patients with rare bleeding disorders undergoing surgery.
• There were very few bleeding events overall with tranexamic acid as the mainstay.
• Both surgery type and plasma coagulation factor level were key in perioperative decision-making.

## 1 Introduction

Rare bleeding disorders (RBDs) are inherited coagulation factor deficiencies, with the exception of hemophilia and von Willebrand disease. The prevalence of homozygous or combined heterozygous forms ranges from 1/300 000 to 1/3 000 000 in function of the concerned factor (F): FII, FV, FVII, FX, FXI, FXIII, or combined FV and FVIII [1]. Due to RBD rarity, many registries have been set up to collect causative mutations, factor levels, and retrospective clinical data [[2], [3], [4]]. These registries are well documented, but often focus on patients with severe deficiencies and very low coagulation factor levels. For patients with severe deficiency (ie, factor level <10%), bleeding severity is strongly correlated with the coagulation factor level, except for FVII and FXI deficiencies, and the treatment guidelines are well codified. Conversely, patients with mild deficiency (factor level >10%) display a wide clinical heterogeneity, even among individuals with the same deficiency, and there is no consensus on their management. Therefore, the health care professionals’ expertise is crucial. As a result, prescribing rules have often been arbitrarily defined according to hypothetical hemostatic thresholds often derived from those used for other more frequent inherited coagulation factor deficiencies, such as hemophilia A and B. Patients with RBDs, often receive factor concentrates or recombinant products, when available, or fresh frozen plasma [5]. However, these factor replacement therapies may be unnecessary and might lead to additional costs or potential immunologic or thrombotic risks. Therefore, treatment guidelines are still needed for RBDs, particularly in the context of surgery.

In patients with RBDs, excessive bleeding during surgery, invasive procedures or childbirth is a feared complication and presurgery bleeding risk stratification is still difficult. Indeed, patients with RBDs cannot be classified only on the basis of the coagulation factor level because of the often-poor relationship between coagulation factor level and bleeding severity, particularly for FVII or FXI deficiency [6]. Therefore, it is also important to collect the detailed bleeding history and to use clinical bleeding scores, such as the International Society on Thrombosis and Haemostasis bleeding assessment tool score developed for von Willebrand disease [7] or the more recent bleeding score adapted to RBDs [8]. Due to RBD rarity, a controlled randomized study would be difficult to conduct.

Here, we performed a multicenter, observational, prospective study to assess the preventive and therapeutic hemostatic approaches adopted before surgery or delivery, and their efficacy in patients with inherited RBDs. The biological (factor levels), clinical (bleeding scores), and surgical (bleeding risk levels) criteria collected were compared with the hemostatic management choice (treatment or only monitoring), and the reported bleeding complications.

## 2 Materials and Methods

### 2.1 Patients

This multicenter, prospective, observational study included 178 patients (61 men, 117 women, M/F sex ratio = 0.52) enrolled at 33 French hemophilia centers from May 2012 to February 2019 (Table 1). The inclusion criteria were: scheduled surgery in patients with low coagulation activity level (<50% for FII, FV, FVII, FX, and FXI; <20% for FXIII; <50% for combined FV and FVIII deficiency). Acquired deficiencies were ruled out by personal and family medical histories, clotting factor inhibitor assays, and in some cases genetic analyses. The study protocol was approved by the National Institutional Review Board comité de protection des personnes (opinion of June 14, 2011 and December 21, 2012), Comité Consultatif sur le Traitement de l’Information en matière de Recherche dans le domaine de la Santé (dossier n. 11.116), and commission nationale de l'informatique et des libertés (declaration n° 1514311).

**Table 1:** Baseline characteristics of the 178 patients included in the study. Due to ethical constraints, race/ethnicity of participants are not available. However, all studied patients live in France and have similar sociocultural determinants.

Factor deficiency	Patients	Sex		Age (y) median (min-max)	Patients with factor level ≤10%	Patients with factor level >10%	Surgeries
FVII	100	M	33	43 (0-84)	15	18	38
		F	67	28 (0-76)	21	46	80
FXI	56	M	21	35 (4-82)	5	16	25
		F	35	31 (14-86)	12	23	39
FII	1	M	1	NA	0	1	1
		F	0	NA	0	0	0
FV	14	M	4	17 (1-31)	1	3	4
		F	10	21 (15-37)	0	10	10
FV+FVIII	3	M	1	NA	1	0	1
		F	2	52 (41-64)	1	1	2
FX	4	M	1	NA	0	1	1
		F	3	51 (34-69)	1	2	6
Total	178	M	61	32 (0-84)	22	39	70
		F	117	31 (0-86)	35	82	137

### 2.2 Data collection

Anamnestic and clinical data were collected before the scheduled surgical procedure using a standardized questionnaire during a face-to-face interview by a specialized hemostasis practitioner. The questionnaire focused on bleeding type and frequency to calculate the Tosetto’s bleeding score [7]. The Tosetto’s bleeding score offers a quantitative evaluation of bleeding symptoms with a “-1” grade for each uneventful hemostatic challenge performed without any replacement therapy and an additional “4” grade of bleeding severity was for each bleeding symptom [7]. It could theoretically range from “-3” (no spontaneous bleeding symptom and no bleeding after surgical procedures) to “+45” (most severe bleed for all symptoms). In the present study, the cut-off value for the bleeding score was chosen to be ≥4, irrespective of gender, since all males and females of the control group in the initial study conducted in 2006 by Tosetto et al. [7] had a score strictly below 4. In addition, all previous surgical procedures were carefully recorded, including bleeding outcomes and the perioperative hemostatic protocol: no procoagulant treatment, factor replacement therapy, and/or tranexamic acid, (TA). Throughout the text, patients are divided in 2 groups: without and with perioperative factor replacement therapy (ie, infusion of recombinant or plasma-derived concentrate of the deficient coagulation factor). Then, data on the bleeding outcome were directly collected by the physician who followed the patient or by contacting the surgery department. Local investigators reported excessive postoperative bleeding as bleeding in the early postoperative period that was greater than expected. However, some of these were reclassified by the authors upon review of individual cases. Excessive postoperative bleeding was defined as bleeding for the type of procedure performed and occurring at the surgical site during the procedure and in the following days. Severe deficiencies were defined by a factor level < ou = to 10%, and all the others as mild deficiencies.

Each invasive procedure was classified as having high or moderate/low intrinsic bleeding risk according to a recent classification [9], except for gynecologic-obstetrical procedures that were categorized by 2 independent physicians, blinded to the results. All surgeries concerning the urinary tract and ear, nose, and throat (ENT) [10] were considered as interventions at sites with high local fibrinolytic activity.

### 2.3 Statistic analysis

The patients’ characteristics are presented using percentages for categorical variables and median (minimum to maximum) for quantitative variables due to their non-Gaussian distribution. Surgical procedures were considered as statistical events. Variables of interest were compared between groups (with vs without perioperative factor replacement therapy) with the Student’s *t* or the Mann–Whiney U-test for continuous variables, and with the Chi-squared or Fisher exact test for categorical variables. An approximate 95% CI was determined for every statistical analysis, and a *P* value < .05 was considered statistically significant. Statistical analyses were performed using SAS 9.1 (SAS Institute, Cary, North Carolina).

## 3 Results

### 3.1 Baseline characteristics

In total, 207 surgical procedures were performed in 178 patients (Table 2). Twelve patients underwent more than one surgical procedure (1-5 procedures). Patients with FVII and FXI deficiency were the most represented: 118 (57.0% of 207) and 64 (30.9%) surgical procedures, respectively (Table 2). Fourteen (6.8%) surgical procedures were performed in patients with FV deficiency, and 11 interventions in patients with FX, FII and combined FV and FVIII deficiency. No surgery concerned patients with FXIII deficiency.

**Table 2:** Surgical procedures and perioperative factor replacement therapy in patients with severe and mild rare bleeding disorders. Only cases in which postoperative bleeding was excessive are listed.

Surgical interventions in patients with severe coagulation factor deficiency (factor level ≤10%).									
Factor deficiency	Total	Perioperative factor replacement	*N* (%)	Factor level median (min-max)	Age (y), median (min-max)	Tosetto’s score, median (min;max)	High risk	Low risk	Surgeries with bleeding (%)
FVII	51	Yes	39 (76.5)	4 (0-10)	48 (0-84)	4 (-1;34)	24	15	1 (2.5)
		No	12 (23.5)	6.5 (2-10)	40 (0-84)	4(-1;7)	3	9	0
FXI	24	Yes	11 (45.8)	4.5 (0-10)	55 (17-86)	1(-2;15)	6	5	1 (9.9)
		No	13 (54.2)	1 (0-10)	31 (22-70)	1(-2;8)	3	10	0
FV	1	Yes	0	NA	NA	NA	NA	NA	NA
		No	1 (100)	NA	NA	NA	0	1	0
FV+FVIII	2	Yes	2 (100)	9 (8-10)	NA	NA	2	0	0
		No	0	NA	NA	NA	NA	NA	NA
FX	3	Yes	2 (66.7)	2 (2-2)	NA	NA	1	1	0
		No	1 (33.3)	NA	NA	NA	0	1	0
Total	81	Yes	54 (66.7)	4 (0-10)	55 (0-86)	4(-2;34)	33	21	2 (3.7)
		No	27 (33.3)	2 (0-10)	35 (0-84)	4(-2;16)	6	21	0


Surgical interventions in patients with mild coagulation factor deficiency (factor level >10%)									
Factor deficiency	Total	Perioperative factor replacement	N (%)	Factor level median (min-max)	Age (y), median (min-max)	Tosetto’s score, median (min;max)	High risk	Low risk	Surgeries with bleeding (%)
FVII	67	Yes	15 (22.4)	18 (12-39)	32 (6-78)	2(-3;12)	10	5	0
		No	52 (77.6)	29 (11-46)	26 (0-78)	3(-3;10)	26	26	1 (1.9)
FXI	40	Yes	8 (20)	29.5 (14-41)	35 (14-74)	2(0;10)	5	3	0
		No	32 (80)	33.5 (14-42)	31 (4-82)	0(-3;6)	17	15	0
FII	1	Yes	0	NA	NA	NA	NA	NA	NA
		No	1 (100)	NA	NA	NA	1	0	0
FV	13	Yes	0	NA	NA	NA	NA	NA	NA
		No	13 (100)	35 (24-47)	21 (1-37)	0(-1;5)	6	7	0
FV+FVIII	1	Yes	0	NA	NA	NA	NA	NA	NA
		No	1 (100)	NA	NA	NA	0	1	0
FX	4	Yes	3 (75)	11 (11-33)	NA	NA	2	1	0
		No	1 (25)	NA	NA	NA	0	1	0
Total	126	Yes	26 (20.6)	23 (11-41)	35 (6-78)	-1(-3;12)	17	9	0
		No	100 (79.4)	31 (11-49)	27 (0-82)	0(-3;10)	50	50	1 (1.0)

### 3.2 Patients with severe factor deficiency

#### 3.2.1 Characteristics of patients without perioperative factor replacement therapy

Among the 81 surgical procedures performed in patients with severe factor deficiency, 27 (33.3%) were performed without perioperative factor replacement (Table 2), particularly tooth extractions (10/13, 76.9%) and gynecologic procedures including delivery (7/19, 36.8%). Conversely, all orthopedic procedures but one (14/15, 93.3%) were performed with perioperative factor replacement therapy. TA was used in most tooth extractions (11/13, 84.6%), in 6/15 (40%) orthopedic surgery, and 5/19 (26.3%) gynecologic/obstetrical procedures (Table 3).

**Table 3:** Perioperative factor replacement therapy in function of the surgery type and the severity of factor deficiency.

Severe deficiencies (coagulation factor level ≤10%)																
	*N*	Orthopedic surgery		TA (%)	Gynecology/ obstetrical surgery		TA (%)	Tooth extraction		TA (%)	Digestive surgery		Maxillofacial and ENT surgery		Miscellaneous surgery	
		*N*	Untreated (%)		*N*	Untreated (%)		*N*	Untreated (%)		*N*	Untreated (%)	*N*	Untreated (%)	*N*	Untreated (%)
All deficiencies	81	15	1 (6.7)	6 (40.0)	19	7 (36.8)	5 (26.3)	13	10 (76.9)	11 (84.6)	10	3 (30.0)	4	2 (50.0)	20	4 (20.0)
F VII	51	9	0	1	11	1	0	7	5	7	8	2	3	2	13	2
F XI	24	5	1	5	8	6	5	4	3	3	1	1	0	0	6	2
F V	1	0	0	0	0	0	0	1	1	0	0	0	0	0	0	0
F V+VIII	2	0	0	0	0	0	0	0	0	0	0	0	1	0	1	0
F X	3	1	0	0	0	0	0	1	1	1	1	0	0	0	0	0
F II	0	0	0	0	0	0	0	0	0	0	0	0	0	0	0	0

Mild deficiencies (coagulation factor level >10%)																
	*N*	Orthopedic surgery		TA (%)	Gynecology/ obstetrical surgery		TA (%)	Tooth extraction		TA (%)	Digestive surgery		Maxillofacial and ENT surgery		Miscellaneous surgery	
		*N*	Untreated (%)		*N*	Untreated (%)		*N*	Untreated (%)		*N*	Untreated (%)	*N*	Untreated (%)	*N*	Untreated (%)
All deficiencies	126	20	13 (65)	4 (20.0)	30	26 (86.7)	11 (36.7)	21	20 (95.2)	16 (76.2)	15	11 (73.3)	15	13 (86.7)	25	18 (72.0)
F VII	67	8	4	1	16^a^	14	6	11	11	6	9	7	8	6	15	11
F XI	40	8	7	3	9	7	5	7	6	7	4	2	5	5	7	5
F V	13	1	1	0	4	4	0	2	2	2	2	2	2	2	2	2
F V+VIII	1	0		0	0		0	1	1	1	0	0	0		0	
F X	4	2	0	0	1	1	0	0	0	0	0	0	0		1	0
F II	1	1	1	0	0		0	0	0	0	0		0		0	

ENT, Ear Nose Throat; Untreated, no perioperative factor replacement (tranexamic acid may have been used)

a All FVII:C levels correspond to baseline levels measured at distance from pregnancy except for one. In this patient FVII:C level was 46%, but the patient was lost to follow-up and the baseline FVII:C level may have been different.

Among the 27 procedures without perioperative factor replacement, 6 were at high bleeding risk: 2 multiple tooth extractions, 1 knee ligamentoplasty, 1 large ethmoidectomy/sphenoidotomy, 1 inguinal hernia surgery, and 1 hysterectomy. Specifically, the multiple tooth extractions, ethmoidectomy, and ligamentoplasty were performed with TA, whereas the hysterectomy and hernia surgery were performed without any perioperative hemostatic treatment. No surgery-related bleeding was reported for these 6 patients among whom 3 with FVII deficiency (FVII:C levels between 5% and 8%) and 3 with FXI deficiency (FXI:C levels between 1% and 2%). The Tosetto’s bleeding score varied from -2 up to 7 (in a patient with multiple tooth extraction).

Among the 6 patients with severe FV, FV and FVIII, or FX deficiency (Table 2), 3 underwent interventions at high bleeding risk that were all managed with factor replacement. The other 3 surgeries were at low bleeding risk and were managed with (*n* = 1) and without (*n* = 2) factor replacement therapy. None of these 6 procedures was complicated by bleeding.

#### 3.2.2 Perioperative factor replacement therapy

Perioperative factor replacement therapy was recorded for 75 surgical procedures performed in patients with FVII deficiency (*n* = 51 interventions) and FXI deficiency (*n* = 24 interventions).

In patients with severe FVII deficiency, 39 of 51 (76.5%) surgeries were managed with recombinant activated factor VII (rFVIIa) (Table 2). Doses ranged from 15 to 30 μg/kg, as recommended by the manufacturer for surgery. For 24 surgical procedures, the same dose was used for all infusions; conversely, a first full-loading dose was followed by half-dose infusions in 11 surgeries. This choice was not influenced by the center or the surgery bleeding risk. Treatment duration varied from 1 to 18 days. The shortest treatment concerned gynecologic surgical procedures by laparoscopy (*n* = 4), one tonsillectomy, and one liver transplantation with one day treatment before clotting factor synthesis recovery. Treatment for orthopedic surgeries (*n* = 5) lasted ≥6 days (Supplementary Table S1A). TA was associated with factor replacement in 9 of 39 (23.1%) surgical procedures, regardless of the surgery bleeding risk (*n* = 5 with high bleeding risk and *n* = 4 with mild bleeding risk) and the surgical site fibrinolytic activity (*n* = 6 with low fibrinolytic activity and *n* = 3 with high activity) (Table 4).

**Table 4:** Tranexamic acid (TA) use (alone or with FR therapy) in the 207 surgical procedures.

Severe deficiencies (coagulation factor level ≤10%)						
Factor deficiency	TA/ all procedures (%)	All procedures	TA alone/ procedures without FR (%)	TA/ all procedures at HFS (%)	High fibrinolytics sites (HFS)	TA alone/ procedures at HFS without FR (%)
		TA and FR/ treated procedures (%)			TA and FR/ treated procedures at HFS (%)	
FVII	17/51 (33)	9/39	8/12	9/11	3/5	6/6
FXI	13/24 (54)	4/11	9/13	3/4	0/1	3/3
FV	1/1 (100)	0	1/1	0/1	0	0/1
FV+FVIII	2/2 (100)	2/2	0	1/1	1/1	0
FX	1/3 (33)	0/2	1/1	1/1	1/1	0
FII	0	0	0	0	0	0
Total	34/81 (42)	15/54 (28)	18/27 (66)	14/18 (78)	5/8 (62)	9/10 (90)

Mild deficiencies (coagulation factor level >10%)						
Factor deficiency	TA/ all procedures (%)	All procedures	TA alone/ procedures without FR (%)	TA/ all procedures at HFS (%)	HFS	TA alone/ procedures without FR (%)
		TA and FR/ treated procedures (%)			TA and FR/ treated procedures at HFS (%)	
FVII	24/67 (36)	3/15	21/52	13/21	2/2	11/19
FXI	25/40 (62)	4/8	21/32	12/12	1/1	11/11
FV	4/13 (30)	0	4/13	3/4	0/0	3/4
FV+FVIII	1/1 (100)	0	1/1	1/1	0/0	1/1
FX	0/4 (0)	0/3	0/1	0	0	0
FII	0/1 (0)	0	0/1	0	0	0
Total	54/126 (43)	7/26 (27)	47/100 (47)	29/38 (76)	3/3 (100)	26/35 (74)

FR, factor replacement; HFS, high fibrinolytics sites; TA, Tranexamic acid.

In patients with severe FXI deficiency, plasma-derived FXI concentrate (pd-FXI, Hemoleven) was used for 11 of 24 (45.8%) surgeries among which 6 were at high bleeding risk. The treatment regimen was heterogeneous (single dose, repeated doses, or one loading dose followed by lower doses) and treatment duration varied from 1 day to ≥6 days (for 3 joint prostheses) (Supplementary Table S1A). TA was associated in 4 surgical procedures at high bleeding risk and low fibrinolytic activity.

For other severe deficiencies, perioperative factor replacement therapy was implemented in 4 surgical interventions: 2 patients with combined FV and VIII deficiency (*n* = 1 intervention/each), and one patient with FX deficiency (*n* = 2 surgeries). TA was used in the 2 patients with combined FV and VIII deficiency who underwent surgical interventions at high bleeding risk of which one with high fibrinolytic activity (tonsillectomy). The tonsillectomy was performed using first fresh frozen plasma (FFP) and TA. Then, following a bleeding episode at day 7 postsurgery, a FVIII concentrate was added for 1 week.

#### 3.2.3 Bleeding outcomes

In patients with severe factor deficiency, excessive bleeding was reported for 5 of 81 (6.1%) surgeries (including one iatrogenic pseudoaneurysm of the radial artery and one spontaneous spleen rupture, without any bleeding complication at the surgery site in both patients). The spleen rupture occurred after left upper lobectomy for lung cancer (Table 5). All 5 interventions were performed with factor replacement therapy (FFP, rFVIIa, or pd-FXI concentrate). Only 2 cases were reclassified as cases in which postoperative bleeding was excessive, one related to the surgery requiring platelet and erythrocyte infusions and one corresponding to a bleed at day 7 after tonsillectomy. In all other cases, postoperative blood loss was considered as expected.

**Table 5:** Characteristics of the patients with excessive bleeding after surgery. All excessive postoperative bleeding events are listed, and the 3 cases in which postoperative bleeding was excessive are highlighted in bold.

Patient (age, y)	Factor level	Procedure	Bleeding score (Tosetto’s)	Perioperative factor replacement	Bleeding	Comments
1 (65)	FVII:C = 1%	Left lung lobectomy	4	rFVIIa	Spontaneous spleen rupture at day 4 postsurgery	No bleeding complication at the lobectomy site
2 (89)	FVII:C = 5%	Nephrectomy	2	rFVIIa	Pseudoaneurysm of the radial artery following catheterization	Cataract surgery performed afterward without factor replacement, no bleeding
3 (79)	FVII:C = 9%	Total knee prosthesis	7	rFVIIa	Bruises at the arms	No bleeding at the surgery site
4 (81)	FXI:C = 10%	Total hip prosthesis	0	pd-FXI (at higher doses than recommended∗)	**Excessive bleeding immediately after surgery and between 24 and 72 h**	Platelet and red blood cell transfusions
5 (13)	FV:C = 10% FVIII:C = 10%	Tonsillectomy	0	FFP and TA	**Excessive bleeding at day 7 postsurgery**	Treatment changed to FVIII concentrate and FFP from day 7 to day 16
6 (34)	FVII:C = 33%	Ectopic pregnancy	10	No treatment	**Hemoperitoneum**	Previous uterine curettage without bleeding and without factor replacement

FVII:C, factor VII activity level; FXI:C, factor XI activity level; pd-FXI, plasma-derived factor XI concentrate; rFVIIa, recombinant activated factor VII; FFP, fresh frozen plasma; TA, tranexamic acid.

### 3.3 Patients with mild factor deficiency

#### 3.3.1 Characteristics of patients without perioperative factor replacement therapy

Among the 126 surgical procedures in patients with mild factor deficiency, 100 (79.4%) were performed without perioperative factor replacement (Table 2). TA was used in 54 of these interventions (43%), particularly for procedures at high fibrinolytic activity sites: tooth extractions (20/21 procedures; 95.2%), and maxillofacial procedures (13/14; 93.3%). This is different from what is observed for surgeries in patients with severe deficiency where TA was used regardless of the surgery bleeding risk and surgical site fibrinolytic activity.

#### 3.3.2 Substitutive treatments

In patients with mild FVII or FXI deficiency, most surgeries were performed without factor replacement therapy (52/67 in patients with mild FVII and 32/40 in patients with mild FXI deficiency) (Table 2). Among the 14 procedures in patients with mild FVII deficiency where rFVIIa was used, 9 were at high bleeding risk. As observed for patients with severe FVII deficiency, 2 regimens were used: one preoperative dose followed by few doses for 2 to 6 days for high bleeding risk surgeries (1 heart and 2 orthopedic surgeries), and a single dose (15 μg/Kg) for 5 procedures classified at low bleeding risk (Supplementary Table S1B). For patients with mild FXI deficiency, a single preoperative dose of pd-FXI was used in 8 interventions, whatever the bleeding risk classification (Supplementary Table S1B). Factor replacement therapy was more frequently used for orthopedic surgical procedures (7/20, 35%) (Table 3).

#### 3.3.3 Bleeding outcomes

Only one bleeding (1/126, 0.8%) was reported as excessive for the 126 surgeries performed in patients with mild factor deficiency. This concerned a woman with mild FVII deficiency (FVII:C = 33%) who underwent surgery for ectopic pregnancy (bleeding score = 10) only with TA. The patient had previously a uterine curettage also performed only with TA without bleeding.

### 3.4 Parameters influencing factor replacement decision-making

Four parameters might influence the choice of perioperative factor replacement therapy: (i) the coagulation factor activity level, (ii) the coagulation factor type, (iii) the surgery type, and (iv) the personal bleeding history or bleeding score.

For all deficiencies, peri-operative replacement therapy was more frequently performed when the factor level was ≤10% (54/81, 66.7%) compared with 11% to 25% (13/33, 39.4%) and 26% to 50% (13/93, 14.0%) (*P* < .0001). Moreover, perioperative replacement therapy was more frequently performed for surgical procedures with high bleeding risk (50/106; 47.2%) than with mild bleeding risk (30/101; 29.7%) (*P = .0099*) (Table 2). Concerning the surgery type (Table 3), orthopedic procedures were more frequently managed with replacement therapy (20/34; 58%), particularly in patients with severe than with mild coagulation factor deficiency (15/16, 93.7% vs 7/20, 35%). The bleeding score also influenced the decision to offer replacement therapy. Indeed, such therapy was more frequent in patients with bleeding score ≥4 (33/56, 58.9%) than <4 (47/151, 31.1%) (*P =* .0004) (Table 6). TA was frequently used (in 88/207, 42.5% of procedures), mainly for surgical procedures at high fibrinolytic sites, such as tooth extractions and ENT procedures (43/56, 76.8%), compared with other sites (45/151, 29.8%) (*P* < .0001). This difference remained significant whatever the severity of the coagulation factor deficiency (Table 4). Indeed, TA was used 2.4-fold and 2.7-fold more frequently for procedures at high fibrinolytic sites than at other sites in patients with severe (14/18 [78%] vs 20/63 [31.7%], *P = .0008*) and also mild deficiency (29/38 [76%] vs 25/88 [28.4%], *P =* .0001).

**Table 6:** Influence of the Tosetto’s bleeding score on perioperative factor replacement decision-making.

	Bleeding score ≤4		Bleeding score >4		*P*
	*n*	Factor replacement (%)	*n*	Factor replacement (%)	
**All factors**	163	53 (32.5)	44	27 (61.4)	<.001
**FVII**	92	36 (39.1)	27	18 (66.7)	.013
**FXI**	56	15 (26.8)	8	4 (50)	.218
**FV**	12	0	2	0	NA
**FX**	2	1 (50)	6	4 (66.7)	NA
**Other**	1	1 (100)	1	1 (100)	NA

NA, not applicable.

The coagulation factor type also influenced the decision of offering replacement therapy as shown by the more frequent administration of coagulation factor concentrates in patients with severe FVII deficiency compared with FXI deficiency (39/51, 76.5% vs 11/24, 45.8%, *P =* .0168). Specifically, FVII deficiency severity and the surgery bleeding risk level were key factors in the replacement therapy decision-making (Figure). A bleeding score ≥4 also was associated with a significantly higher rate of replacement therapy (21/33, 63.6% vs 32/83, 38.6% respectively; *P = .0224*). In FXI deficiencies, these 3 parameters also were important for replacement therapy decision-making, but their association was not significant as shown in Figure and the absence of significant difference in the percentage of treated patients with bleeding score ≥4 (6/12, 50%) vs <4 (13/52, 25%) (*P =* .1574).

**Figure:**
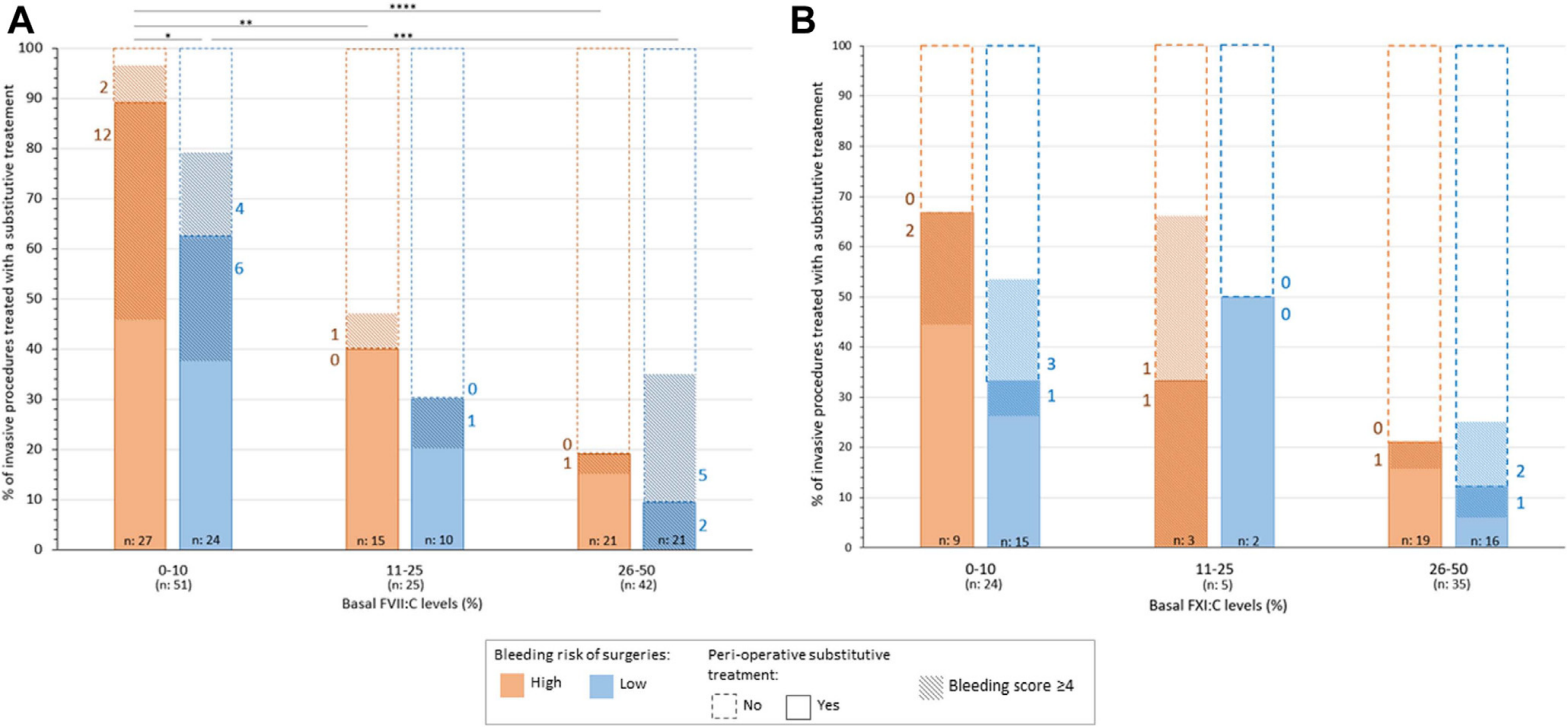
Influence of the basal coagulation factor level, surgery bleeding risk, and bleeding score on perioperative factor replacement decision-making in patients with FVII (A) and FXI deficiency (B). BR = bleeding risk. The total number of surgical procedures is indicated at the bottom of each bar. The number of patients with a bleeding ≥4 is indicated on the side of each hatched bar. ∗∗∗∗*P* < .00001, ∗∗∗.00001 ≤ *P* < .0001, ∗∗.0001 ≤ *P* < .001, ∗.001 ≤ *P* < .05.

## 4 Discussion

In this study, we report a prospective analysis of 207 minor and major surgical procedures performed in 178 patients with a RBD. We wanted to evaluate the perioperative hemostatic management (factor replacement, TA) by experts at 33 French centers, and specifically the criteria that might have driven their choice. The ultimate goal was to contribute to future guidelines for perioperative factor replacement in RBDs in function of the coagulation factor level and surgery type. As bleeding events were very rare (6/207), even for surgical interventions performed without perioperative factor replacement therapy, we focused on the procedures performed without factor replacement to better understand their hemostatic management (eg, TA, only monitoring).

Among the 6 postsurgery bleeding episodes, only 3 were considered as excessive surgery-related bleeds: (i) a total hip prosthesis in a patient with FXI deficiency supplemented with high doses of pd-FXI who required the transfusion of platelets and red blood cells; (ii) a tonsillectomy in a child with combined FV and VIII severe deficiency, followed by bleeding at day 7 postsurgery and treated with FFP and TA; and (iii) a woman with mild FVII deficiency and surgery for an ectopic pregnancy without any factor replacement therapy resulting in hemoperitoneum. The other 3 bleedings were not at the surgery site but due to (i) spontaneous spleen rupture (ii) bruises at the injection point in the arm, and (iii) distal pseudoaneurysm due to radial artery catheterization. Spleen rupture occurred in a 65-year-old patient at day 4 after a left upper lobectomy. Atraumatic spleen rupture in relation with lung surgeries are usually consecutive to splenic metastasis [11], but there was no pathological evidence of this in our patient. Moreover, during the 4 days between surgery and spleen rupture, no blood-stained fluid in the drainage tube was observed. Therefore, this bleeding event was not considered as an excessive bleed linked to the surgery. In all other cases, postoperative blood loss was as expected and no other adverse event was reported. This very low number of hemorrhagic events could be explained by the fact that each intervention was performed in close collaboration with hemostasis specialists who followed the currently available protocols. This underlines the importance of multidisciplinary consultations involving surgeons, anesthetists, and hemostasis specialists. Indeed, for the 80 procedures with perioperative factor replacement therapy, the therapeutic regimens were fairly homogeneous and according to guidelines [[12], [13], [14], [15], [16], [17]]. Indeed, the therapeutic management of the included patients was supervised by experts in centers specialized in hemostasis disorders confirming the robustness of the published guidelines [18]. Moreover, it emphasizes the point that whenever possible, surgery in patients with RBDs should be performed at centers with specialized expertise, namely *hemophilia treatment centers*, or at least in consultation with such centers.

It could be interesting to analyze the criteria that might have driven the therapeutic choice to treat or not to treat. Three main criteria were evaluated: surgery type, coagulation factor activity, and bleeding history (ie, Tosetto’s bleeding score prospectively recorded). All 3 criteria played a significant role (Figure, Table 5). The coagulation factor level influenced the choice of perioperative factor replacement in both patients with FVII and FXI deficiency undergoing high bleeding risk surgeries, but only in patients with FVII deficiency for low bleeding risk surgeries. The high proportion of procedures with perioperative factor replacement in patients with high FXI:C levels (Figure) is in accordance with literature data showing a poor relationship between FXI level and bleeding risk after surgery [6,19]. However, factor level measurement was performed in each center and this might have introduced some accuracy heterogeneity, especially for low values, compared with results obtained in a centralized laboratory. The accuracy of the FVII:C levels is another limitation of our study for 2 reasons. First, the species-derived thromboplastin preparation used to measure the FVII:C levels could not be identified for each patient. Therefore, some FVII variants could have led to FVII:C value underestimation when measured with thromboplastin of rabbit origin. Second, it is known that FVII:C levels vary throughout women’s life [20,21]. In their recent review, Abdul-Kadir et al. [22] reported the possibility of a modest FVII:C level increase during pregnancy, but only in women heterozygous for FVII causative variants, while women homozygous for these variants generally do not show this increase. However, in our study, only in one patient the FVII:C level measurement of 46% overlapped with pregnancy, thus potentially leading to FVII:C overestimation.

Concerning the surgery type, high bleeding risk procedures frequently led to perioperative factor replacement therapy. However, in patients with severe RBD, 27 surgical procedures were performed without factor replacement among which 6 were at high bleeding risk (Table 2), including one hysterectomy, one large ethmoidectomy/sphenoidotomy, and one knee ligamentoplasty that are considered at very high risk of bleeding. Analysis of the data on these 6 untreated patients showed that 3 high bleeding risk surgical procedures were performed in 3 patients with very low FXI:C level (<2%), in line with the fact that FXI level alone is probably not sufficient for perioperative factor replacement decision-making. In these 3 patients, the Tosetto’s bleeding score (-2, 0, and 1, respectively) seemed to be the main criterion driving the practitioner’s therapeutic decision. In patients with FXI deficiency, orthopedic procedures and appendicectomy usually do not require factor replacement [15,16]. The other 3 high-risk surgeries performed without factor replacement concerned patients with severe FVII deficiency (FVII:C level between 5% and 8%, but below the previously published hemostatic thresholds of 8.5%, 15%, or 20% [2,23,24]). In these 3 patients, the Tosetto’s bleeding score was between 5 and 7, much higher than the threshold of 4.

Similarly, 7 surgeries were performed without any perioperative factor replacement therapy in 7 patients with mild FVII deficiency (FVII:C level >20%) and Tosetto’s score between 5 and 10 (Supplementary Table S2). Among them, a patient with hemorrhage after surgery for ectopic pregnancy had Tosetto’s score of 10 and a FVII:C level of 33%. Literature data indicate that the hemorrhagic clinical phenotype is the criterion best correlated with the bleeding risk [25]. However, criticisms about the reliability of bleeding scores have emerged because they were developed for more frequent bleeding disorders and not for RBDs. In 2016, Palla et al. [8] developed a bleeding score for RBDs that could not be evaluated in this study which started before the Palla and coworkers’ publication.

Considering the other RBDs, all procedures performed in patients with FX deficiencies were associated with factor replacement therapy except 2: one tooth extraction performed only with TA and a delivery in a woman with FX:C level of 40% but Tosetto’s score of 10. In patients with FV deficiency, the FV:C level was >20% and most surgeries were performed without perioperative factor replacement. Only one (tooth extraction) concerned a patient with severe FV deficiency (FV:C level of 2%) and Tosetto’s score of 0. None of these patients reported bleeding complications.

In summary, despite the poor relationship between coagulation factor levels and bleeding symptoms, the factor level seems to be the most important parameter influencing the perioperative factor replacement decision-making in patients with FVII or FX deficiency, whereas it is less important for patients with FXI deficiency for whom the surgical site and the clinical phenotype seem to be the best criteria.

This study also provides information on alternative treatments, such as TA. TA is commonly used for surgeries at mucosal sites and at high fibrinolytic activity tissues [10]. In our study, it was used in 42% of all procedures and in 51.2% of procedures without factor replacement. Importantly, TA was used in 59.4% of procedures in patients with FXI deficiency, despite no clear guidelines about TA in this population (Table 4). In patients with FXI deficiency, only expert opinions suggest TA alone for minor surgery, and alone or in combination with a low dose of rFVIIa or FFP for major surgery [26,27]. This high rate of TA prescriptions by French practitioners is probably explained by the known physiological role of FXI in fibrinolysis down-regulation [28]. Indeed, reduced fibrinolytic resistance was observed in patients with FXI deficiency in whom the thrombin-activable fibrinolysis inhibitor pathway was impaired [29]. Furthermore, in our cohort, TA was used in about one third of surgeries performed at mucosal sites (eg, ears, nose, throat, uterus, and cervix) as proposed for hereditary coagulopathies [30]. In our cohort, procedures performed with TA alone or associated with coagulation factor concentrates were without bleeding or adverse events. Although these data do not allow drawing conclusions on TA efficacy, they provide arguments for the safe use of TA for procedures at mucosal sites in patients with inherited RBDs and coagulation factor levels below the threshold for bleeding risk, especially FXI deficiency.

In conclusion, our findings indicate that in a multidisciplinary approach that implicates surgeons, anesthetists, and hemostasis experts, the hemostatic management of a patient with RBD undergoing surgery is based mainly on the coagulation factor level (less obvious for FXI) and the surgery type/bleeding risk rather than on the patient’s clinical history and bleeding score. The next major question concerns the best time for factor replacement therapy if required: (i) preventively for surgery in areas that are impossible or difficult to access for hemostasis procedures, or (ii) “on demand” in case of bleeding. On the other hand, this prospective study confirms that surgical procedures can be safely performed without factor replacement therapy in many patients with RBDs.
