# Supplementary material for: Surgery in rare bleeding disorders: the prospective MARACHI study
Source: Res Pract Thromb Haemost. 2023 Sep 6;7(7):102199. doi: 10.1016/j.rpth.2023.102199 (PMC10585326; doi:10.1016/j.rpth.2023.102199)
Supplement: Supplementary material [file mmc1.pdf]

Table S1: Perioperative factor replacement regimens used in patients with FVII and FXI deficiency (factor level ≤10%) in function of the surgery bleeding risk. A) Severe FVII and FXI deficiencies (factor level ≤10%). B) mild FVII and FXI deficiencies. FFP was given to treat a spontaneous spleen rupture after lung surgery, but not as first-line treatment (Patient 1, Table 5)

Table S1A

**High bleeding risk surgical procedures**				
**FVII;** recombinant FVIIa (n=24)			**FXI;** plasma-derived FXI (n=6)	
**Therapeutic regimen**	**n=**	**Mean doses µg/kg (min-max)**	**n=**	**Mean doses IU/kg (min-max)**
Single dose	2	31.5 (30-33)	1	22
	(+FFP) 1	25.0	0	
Same dose repeated	13	25.2 (12-33)	2	19.5 (13-26)
1 loading dose followed by lower doses	7	26.3 (22-36)<br>12.9 (11-18)	3	26.7 (25-30)<br>11.3 (10-12.5)
1 preoperative dose followed by continuous infusion	1	Bolus : 25.0 then 90.0 per day	0	
**Treatment duration**	**n=**	**Comments**	**n=**	**Comments**
1 day	6	1 or several injections	1	
2 to 5 days	7	2 total knee prosthesis (treated for 4 days)	2	1 cardiac surgery<br>1 orthopedic surgery
≥ 6 days	11	5 orthopedic surgeries	3	3 joint prosthesis (knee/hip)
**Low/moderate bleeding risk surgical procedures**				
Recombinant FVIIa (n=15)			Plasma-derived FXI (n=5)	
**Therapeutic regimen**	**n=**	**Mean doses µg/kg (min-max)**	**n=**	**Mean doses IU/kg (min-max)**
Single dose	7	22.4 (15-33)	5	15.0 (11-17)
Same dose repeated	4	23.8 (13-30)	0	
1 loading dose followed by lower doses	4	24.3 (19-31)<br>13.8 (9,5-21)	0	
**Treatment duration**	**n=**	**Comments**	**High fibrinolytic activity**	**Mean doses IU /kg**
1 day	9	1 injection n=7<br>2 injections n=2	n=1	16.0
2 to 5 days	3			
≥ 6 days	3	3 obstetric procedures		

Table S1B.

High bleeding risk surgical procedures				
**FVII;** recombinant FVIIa (n=9)			**FXI;** plasma-derived FXI (n=5)	
**Therapeutic regimen**	**n=**	**Mean doses µg/kg (min-max)**	**n=**	**Mean doses IU /kg**
Single dose	3	12.3 (10-14)	5	15.6 (11-28)
Same dose repeated	4	21.3 (14-31)	0	
1 loading dose followed by lower doses	2	25.0 (20-30)<br>14.5 (14-15)	0	
**Treatment duration**	**n=**	**Comments**	**n=**	**Comments**
1 day	5	1 treatment by FFP	5	
2 to 5 days	2	ENT surgeries	0	
≥ 6 days	3	2 orthopedic surgeries<br>1 cardiac surgery	0	
**Low/moderate bleeding risk procedures**				
**FVII;** recombinant FVIIa n=5			**FXI;** plasma-derived FXI (n=5)	
**Therapeutic regimen**	**n=**	**Mean doses µg/kg (min-max)**	**n=**	**Mean doses IU /kg (min-max)**
Single dose	5	22.0 (16-50)	3	12.7 (10-15)
**Treatment duration**	**n=**	**Comments**	**High fibrinolytic activity**	
1 day	5		n=1	15.0

Table S2: Description of patients with bleeding score >4

Patients	Factor VII:C level	Intervention number	Procedure	Bleeding score (Tosetto's)	Perioperative factor replacement	Excessive bleeding	Tranexamic acid
BO-A	38	Intervention n. 1	Removal of bilateral abscess (Verneuil's disease)	5	Yes	No	No
CH-M	44	Intervention n. 1	Varicose vein stripping	5	No	No	No
KO-A	37	Intervention n. 1	Extraction of 4 wisdom teeth	6	No	No	No
RE-J	28	Intervention n. 1	Hymen surgery	6	No	No	Yes
TH-C	33	Intervention n. 1	Hepatic cyst surgery by laparoscopy	6	Yes	No	No
LE-A	48	Intervention n. 1	Vaginal delivery	7	No	No	No
MA-N	33	**Intervention n. 1**	Uterine curettage	8	No	No	Yes
		**Intervention n. 2**	Hemoperitoneum after extrauterine molar pregnancy	10	No	Excessive bleeding	Yes
RI-S	20	Intervention n. 1	Ablation of tumor in the right breast and of sentinel node	8	No	No	No
DU-V	39	Intervention n. 1	Removal of cysts in right and left tibias and right elbow	12	Yes	No	Yes
**Patients**	**Factor XI:C level**	Intervention number	**Procedure**	**Bleeding score** (Tosetto's)	**Perioperative factor replacement**	**Excessive bleeding**	**Tranexamic acid**
BE-E	36	Intervention n. 1	Dental extraction (molar)	5	No	No	Yes
CH-P	30	Intervention n. 1	Cholecystectomy by laparoscopy	5	Yes	No	No
FO-A	31	Intervention n. 1	Childbirth	5	No	No	Yes
GU-G	22	Intervention n. 1	Right hip total prosthesis	6	No	No	Yes
DE-O	26	Intervention n. 1	Wisdom tooth extraction	10	Yes	No	Yes
**Patients**	**Factor X:C level**	Intervention number	**Procedure**	**Bleeding score** (Tosetto's)	**Perioperative factor replacement**	**Excessive bleeding**	**Tranexamic acid**
FO-J	40	Intervention n. 1	Vaginal delivery	10	No	No	No
**Patients**	**Factor V:C level**	Intervention number	**Procedure**	**Bleeding score** (Tosetto's)	**Perioperative factor replacement**	**Excessive bleeding**	**Tranexamic acid**
AB-C	28	Intervention n. 1	Extraction of 4 wisdom teeth	5	No	No	Yes
AI-M	30	Intervention n. 1	Rhinoseptoplasty	5	No	No	Yes
